# Dynamically Reconfigurable XNOR/IMP Logic Based on Dual-Mechanism Operation in an Electrically Tunable Two-Dimensional Heterojunction

**DOI:** 10.3390/nano16050335

**Published:** 2026-03-09

**Authors:** Yuting He, Jinbao Jiang, Feng Xiong, Zhihong Zhu

**Affiliations:** 1 College of Advanced Interdisciplinary Studies & Hunan Provincial Key Laboratory of Novel Nano-Optoelectronic Information Materials and Devices, National University of Defense Technology, Changsha 410073, China; 17352889419@163.com (Y.H.); jiangjinbao@foxmail.com (J.J.); xiongfeng11@nudt.edu.cn (F.X.); 2 Nanhu Laser Laboratory, National University of Defense Technology, Changsha 410073, China

**Keywords:** reconfigurable logic, electrically tunable, Fowler–Nordheim tunnel

## Abstract

Reconfigurable logic is crucial for future adaptive computing, but is challenging to realize with conventional complementary metal-oxide-semiconductor technology due to the limited field-effect characteristics of the fundamental silicon devices. Two-dimensional materials offer a promising platform, yet enhancing their functional versatility requires novel operational mechanisms. Here, we demonstrate a single WSe_2_/h-BN/graphene heterojunction capable of dynamically switching between distinct logic functions—XNOR and IMP (implication gate or “IF-THEN” gate)—simply by modulating the drain-source voltage. At a low bias of 0.3 V, the carrier distribution is governed by capacitive coupling, realizing an XNOR gate. Increasing the bias to 3 V activates Fowler–Nordheim tunneling between the graphene floating gate and the drain, enabling IMP logic operation. The interplay and voltage-induced transition between these two physical mechanisms underpin the device’s multifunctional capability. This work introduces a novel operational strategy for two-dimensional material-based reconfigurable logic, providing a pathway toward compact, adaptive hardware for post-CMOS computing.

## 1. Introduction

The miniaturization of conventional metal-oxide-semiconductor field-effect transistors (MOSFETs) is approaching its physical limits [1]. To further improve information processing performances, electronic devices based on new physical principles or emerging technologies are required [2,3,4]. In this context, reconfigurable logic has emerged as a transformative paradigm, offering the ability to dynamically alter circuit functionality within the same hardware footprint [5,6,7,8,9,10]. Such capability is crucial for adapting to diverse computational tasks, enhancing hardware utilization, and enabling more flexible and compact computing systems, which are central to the development of adaptive electronics, edge computing, and neuromorphic engineering [11,12].

The advent of two-dimensional (2D) materials has opened new avenues for such innovations [10]. Materials like graphene, transition metal dichalcogenides, and hexagonal boron nitride exhibit exceptional electronic, optical, and mechanical properties, along with atomically thin bodies that enable potent electrostatic control [13,14,15,16,17]. Over the past decade, research into 2D material-based transistors and logic devices has intensified, demonstrating a promising pathway toward more sophisticated, functionally integrated systems [9,11,18,19,20,21,22,23,24]. Notably, these include the development of neuromorphic computing components that mimic synaptic and neuronal functions for energy-efficient, brain-inspired hardware [8,25,26]; devices capable of synergistic optoelectronic co-modulation, where optical and electrical inputs interact to define novel operational states [18,27,28,29,30,31,32]; and architectures that integrate logic and non-volatile memory functions (logic-in-memory)—to overcome the von Neumann bottleneck [33,34,35,36,37]. Collectively, these advances underscore a shift from simple electronic switches toward reconfigurable, multi-domain systems with broader application potential. Despite these advances, the versatility of 2D logic devices can be further enhanced by introducing novel operational mechanisms, thereby enabling distinct reconfigurable logic from a single device.

Here, we demonstrate a WSe_2_/h-BN/graphene heterostructure that exhibits dynamically reconfigurable multifunctional logic operations (XNOR and IMP), controlled by the polarity combinations of gates and drain voltages. The ambipolar WSe_2_ channel is simultaneously modulated by the floating gate and the back gate, where the floating-gate voltage regulates the channel regions on both sides of the electrode, while the back-gate voltage governs the central area of the channel. Under a low source-drain bias (0.3 V), the electrostatic coupling from both the floating gate and the back gate primarily dictates the carrier distribution within the channel, enabling the realization of the XNOR logic function. When the source-drain bias is increased (3 V), the operational mechanism is input-dependent. For the (0, 0) and (0, 1) input combinations, Fowler–Nordheim tunneling between the floating gate and the drain dominates, while for (1, 0) and (1, 1), electrostatic coupling remains the primary mechanism. IMP logic is therefore realized through the coexistence of these two mechanisms. The coexistence and interplay of these two mechanisms underpin the device’s multifunctional capability. By introducing a new mechanistic dimension into 2D device operation, this work provides a viable pathway toward more versatile and compact post-CMOS computing hardware.

## 2. Materials and Methods

**Figure 1:**
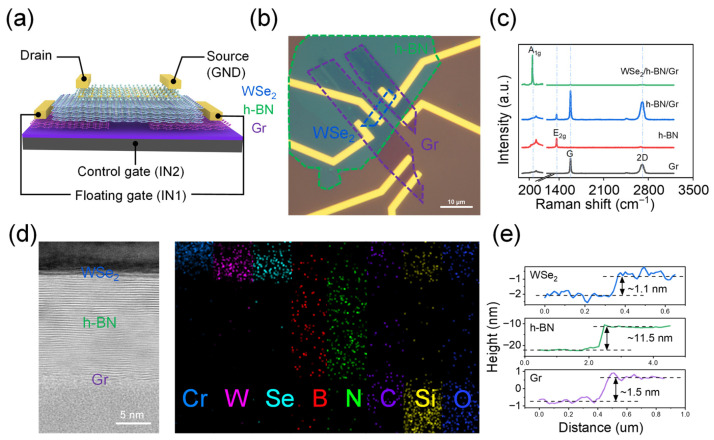
Schematic illustration of the WSe_2_/h-BN/Gr heterojunction. (**a**) Schematic diagram of the WSe_2_/h-BN/Gr heterojunction and the corresponding circuit configuration; (**b**) optical microscope image of the fabricated WSe_2_/h-BN/Gr heterojunction; (**c**) Raman spectroscopy of the Gr flakes, h-BN flakes, h-BN/Gr heterostructure, and WSe_2_/h-BN/Gr heterostructure; (**d**) cross-sectional TEM image and corresponding energy-dispersive spectrometry (EDS) of WSe_2_/h-BN/Gr heterostructure beneath the source electrode; (**e**) the atomic force microscopy (AFM) results of WSe_2_, h-BN, and Gr flakes.

A schematic diagram of the heterojunction fabricated on a commercial SiO_2_/Si wafer is depicted in Figure 1a, where tungsten diselenide (WSe_2_), hexagonal boron nitride (h-BN), and graphene (Gr) flakes serve as the channel layer, tunneling dielectric, and floating gate, respectively. Two graphene floating gates (FG) are placed beneath the source and drain electrodes, respectively, and connected together to serve as input 1 (IN1). Notably, “floating gate” (FG) terminology is adopted primarily to describe the device structure, and that the graphene layer in this work functionally serves as a middle gate rather than a conventional floating gate for charge storage. The heavily doped silicon back gate (BG) acting as the input 2 (IN2). Figure 1b shows the optical microscope image of the photodetector, where WSe_2_, h-BN, and Gr flakes are indicated by blue, green, and purple dashed lines, respectively. As shown in the figure, the channel length between the source and drain is approximately 6.5 μm. The Raman spectroscopy of the Gr flakes, h-BN flakes, h-BN/Gr heterostructure, and WSe_2_/h-BN/Gr heterostructure are depicted in Figure 1c. The WSe_2_ flakes exhibit one characteristic peak at 254 cm^−1^, corresponding to the out-of-plane (*A*_1g_) vibrational modes. The characteristic peak of h-BN is located at 1366 cm^−1^, corresponding to the in-plane (*E*_2g_) vibrational mode. Furthermore, the peaks at 1578 cm^−1^ (*G*) and 2705 cm^−1^ (2*D*) are two typical characteristic peaks of multi-layer graphene. The cross-sectional TEM image and energy-dispersive spectrometry (EDS) of WSe_2_/h-BN/Gr heterostructure beneath the source electrode is obtained and the results are shown in Figure 1d. The boundary of h-BN/Gr and WSe_2_/h-BN interfaces can be clearly resolved, and the thicknesses of WSe_2_, h-BN, and Gr flakes is identified to be 1.1, 11.5 and 1.5 nm, respectively, in good accordance with the atomic force microscopy (AFM) results in Figure 1e.

The heterostructure was constructed with the standard dry transfer method. The commercial SiO_2_/Si substrate (285 nm oxide thickness) first underwent a standard cleaning procedure. It was immersed sequentially in acetone, isopropanol (IPA), and deionized (DI) water, followed by drying with a nitrogen stream to obtain an atomically clean surface. Subsequently, flakes of multilayer graphene, h-BN, and WSe_2_ were transferred onto the pre-cleaned SiO_2_ surface via mechanical exfoliation. The PC adhesion layer can pick up 2D materials at ~90 °C and release them at ~180 °C, and the WSe_2_ h-BN flakes were sequentially picked up by this polymer and finally released onto the graphene flake on the SiO_2_/Si substrate [38]. The remaining polycarbonate (PC) was dissolved in a chloroform (CHCl_3_) bath for two minutes. Subsequently, the device was annealed at 300 °C under vacuum for three hours to improve interfacial contact and release residual stress within the heterostructure. Finally, electrodes were defined via standard electron-beam lithography (EBL) and deposited by electron-beam evaporation of Cr/Au (5/50 nm).

## 3. Results and Discussions

**Figure 2:**
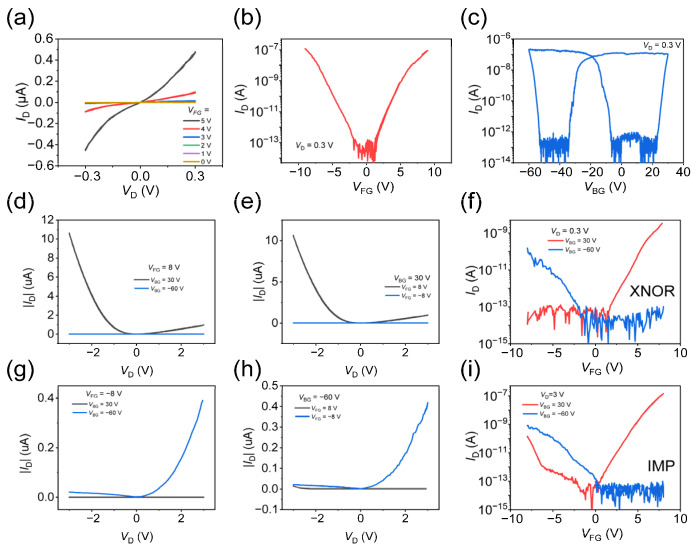
Transport properties of the device. (**a**) Output curves under various floating-gate voltage; (**b**) transfer curves under the modulation of floating gate at 0.3 V bias; (**c**) transfer curves under the modulation of back gate at 0.3 V bias; (**d**,**g**) IV curves with floating gate at 8/−8 V and back gates at 30 V and −60 V; (**e**,**h**) IV Curves with back gate at 30/−60 V and floating gates at 8 V and −8 V; (**f**) transfer curves under the modulation of floating gate at 0.3 V bias and back gates at 30 V and −60 V; (**i**) transfer curves under the modulation of floating gate at 3 V bias and back gates at 30 V and −60 V.

The electrical properties of the WSe_2_ layer were first characterized by I–V and transfer curve measurements. Figure 2a presents the I–V characteristics of the WSe_2_/h-BN/Gr heterostructure under various floating-gate voltages. (Schematic of the measurement circuit for Figure 2a–c is illustrated in Figure S1). Within the low-bias regime, the curves exhibit a nearly linear behavior, indicating the formation of a quasi-Ohmic contact between the source/drain electrodes and the WSe_2_ channel. This linearity confirms a low injection barrier and high-quality electrical contact at the electrode/channel interfaces, which lays a reliable foundation for the subsequent logic operations. Figure 2b shows the transfer curve of WSe_2_ at a source-drain voltage of 0.3 V under floating-gate modulation. The curve exhibits ambipolar behavior with a high on/off ratio of up to 10^6^, demonstrating effective control of WSe_2_ by the floating gate. Figure S4a shows the bidirectional transfer curves under 0.3 V and 3 V bias, which exhibit a negligible hysteresis window across the entire scanned voltage range, indicating the high quality of the WSe_2_/h-BN/Gr heterostructure and negligible charge trapping/de-trapping on the interfaces. Figure 2c shows the transfer characteristics measured under back-gate modulation with the FG left floating. A clear memory window is observed, which results from charge storage in the floating gate when it is not connected to an external circuit [39,40]. However, in all subsequent measurements, the FG is directly connected to an external electrode, representing a different operating condition. This figure is included solely to demonstrate the back gate’s capability to modulate the channel. Overall, the WSe_2_ demonstrates effective and independent modulation by both the floating gate and the back gate. In addition, the WSe_2_ channel exhibits clear ambipolar conduction, and the maximum on-state current occurs at a floating-gate voltage of 8 V (back-gate voltage of 30 V) for electron-dominated conduction and −8 V (back-gate voltage of −60 V) for hole-dominated conduction.

As shown in Figure 1a, the device’s channel is electrostatically controlled by both the floating gate and the back gate, with the FG dominating near the electric contacts and the BG in the mid-channel. High conduction is achieved only when both gates accumulate carriers of the same type (both electrons or holes). When both gates bias the channel toward electron/hole accumulation, a uniform conductive path forms, turning the device on. Conversely, a mismatch in carrier type—where one gate induces electron conduction and the other induces hole conduction—creates a potential barrier or carrier depletion in the channel, thus leading to a significantly reduced drain current. Figure 2d,g present the I–V characteristics measured at a floating-gate voltage of 8 V/−8 V, with the back-gate voltage set to 30 V and −60 V. Similarly, Figure 2e,h demonstrate the I–V characteristics under a constant BG voltage of 30 V (−60 V), with the FG voltage set to 8 V and −8 V. A key observation from the figures is that the channel current is significantly larger when the floating-gate and back-gate voltages have the same polarity, compared to when they have opposite polarities. The source-drain current with *V*_FG_ = 8 V (−8 V) and *V*_BG_ = 30 V (−60 V) is substantially higher than that with *V*_FG_ = 8 V (−8 V) and *V*_BG_ = −60 V (30 V), highlighting the critical role of gate voltage alignment.

Furthermore, the drain current increases with the applied source-drain voltage, as expected. Figure 2f and Figure 2i present the corresponding transfer curves measured at source-drain voltages of 0.3 V and 3 V, respectively. At *V*_D_ = 0.3 V, the transfer curve exhibits n-type semiconductor behavior when *V*_BG_ = 30 V, while exhibiting p-type behavior when *V*_BG_ = −60 V. This indicates that back-gate voltage determines the type of majority carrier induced in the channel layer. By applying floating-gate voltage and back-gate voltage as inputs, the device can be configured to function as an XNOR logic gate. In contrast, at *V*_D_ = 3 V, a distinct ambipolar behavior is observed in the transfer characteristics when *V*_BG_ = 30 V, while the transfer curve maintains p-type behavior when *V*_BG_ = −60 V. The underlying mechanism for this ambipolar behavior is the strengthening of the electric field between the floating gate and the drain with increasing drain bias. This enhanced field gives electrons a higher probability of tunneling from the floating gate to the drain, thereby inducing n-type characteristics in the channel region near the drain, similar to the central region [41]. By utilizing the electron tunneling effect, the device can be configured to function as an IMP (implication) logic gate, with floating-gate voltage and back-gate voltage as inputs. IMP (implication) logic, often expressed as “P IMPLIES Q” (P → Q), is a fundamental Boolean operation where the output is false only when the first input (P) is true and the second input (Q) is false. Otherwise, the output is true [42].

**Figure 3:**
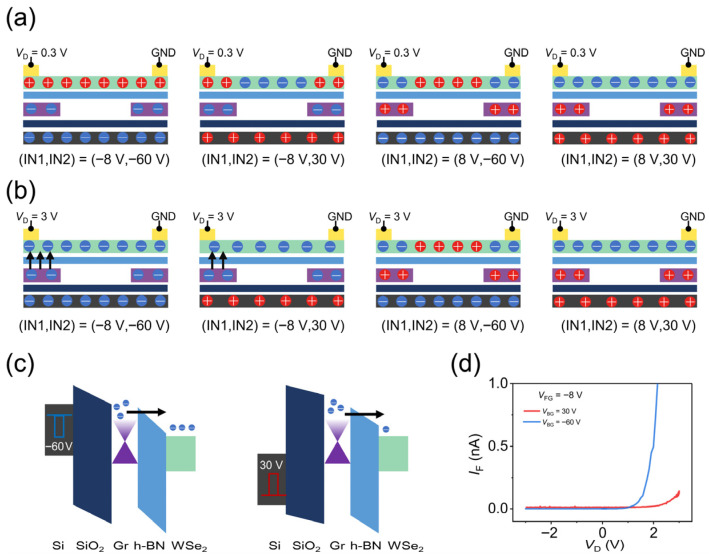
Working principle of the reconfigurable logic device. (**a**,**b**) Schematic illustration of charge carrier distribution at 0.3 V and 3 V bias, respectively. The red circles represent holes, and the blue circles represent electrons. From top to bottom, the device is structured as WSe_2_, h-BN, Gr, SiO_2_, and Si. The solid arrow indicates the direction of electron injection; (**c**) energy band diagram for electron tunneling at −60 V and 30 V back-gate voltage; the floating-gate voltage is −8 V, and the V_D_ is 3 V; (**d**) tunneling current as a function of V_D_ with the floating-gate voltage set to −8 V and back-gate voltage set to −60 V/30 V.

To elucidate the reconfigurable logic functionality of the device, we investigate its operating mechanism by examining the charge carrier distribution and energy band structure. The charge carrier distribution through the WSe_2_ channel is jointly modulated by the silicon back gate (*V*_BG_), the graphene floating gate (*V*_FG_), and the drain-source bias (*V*_D_). We take the device under a small *V*_D_ (0.3 V) as a detailed example to illustrate how floating-gate voltage (IN1) and back-gate voltage (IN2) affect the charge carrier distribution. As shown in Figure 3a, when the *V*_FG_ and *V*_BG_ are set to −8 V and −60 V ((IN1, IN2) = (−8 V, −60 V)), a negative voltage applied to the floating gate induces hole accumulation in the channel regions near the source and drain electrodes due to electrostatic induction. Similarly, a negative voltage applied to the back gate leads to hole accumulation in the central region of the channel. (Due to electrostatic screening from the floating gate, the back-gate capacitive coupling effect is largely restricted to the central part of the channel). Consequently, the entire channel exhibits p-type conduction behavior. Conversely, when the *V*_FG_ and *V*_BG_ are set to −8 V and 30 V ((IN1, IN2) = (−8 V, 30 V)), the negative floating-gate voltage still causes hole accumulation near the electrodes, while the positive back-gate voltage induces electron accumulation in the middle section of the channel. This results in a p-n-p configuration across the channel. The formation of the central electron-potential well significantly suppresses the source-drain current. The charge distributions for the remaining cases, derived from the same analysis, are also presented in the figure. The results demonstrate that the channel conduction is uniformly n-type (when (IN1, IN2) = (8 V, 30 V)) or p-type (when (IN1, IN2) = (−8 V, −60 V)) when the floating-gate and back-gate voltages share the same-polarity, whereas opposite polarities induce either an electron ((IN1, IN2) = (−8 V, 30 V)) or hole ((IN1, IN2) = (8 V, −60 V))potential well, leading to a significantly suppressed source-drain current. This observation is fully consistent with the transport properties presented earlier.

When the drain-source voltage is set to 3 V (Figure 3b), the device behavior differs under a floating-gate voltage of −8 V. At *V*_D_ = 3 V and *V*_FG_ = −8 V, the electric field across h-BN becomes sufficiently strong to induce tunneling. Consequently, the floating gate modulates the channel carrier distribution predominantly through direct charge injection instead of capacitive coupling. Figure 3c presents the energy band diagrams of the device [43] under a source-drain voltage of 3 V and a floating-gate voltage of −8 V, with the back-gate voltage set to −60 V and 30 V, respectively. A back-gate voltage of −60 V generates a stronger electric field in the h-BN tunneling layer, leading to more pronounced band bending. This stronger band bending facilitates greater electron tunneling into the channel. In contrast, at *V*_BG_ = 30 V, the electric field induced by the back gate in h-BN opposes that of the floating gate, thereby partially canceling the overall field and resulting in weaker band bending and thus less electron tunneling. The corresponding tunneling currents are quantified in Figure 3d, measured at *V*_FG_ = −8 V for *V*_BG_ = 30 V/−60 V. It is evident that the tunneling current at *V*_BG_ = −60 V is significantly larger than that for *V*_BG_ = 30 V. This experimental result directly corroborates the theoretical analysis presented above.

**Figure 4:**
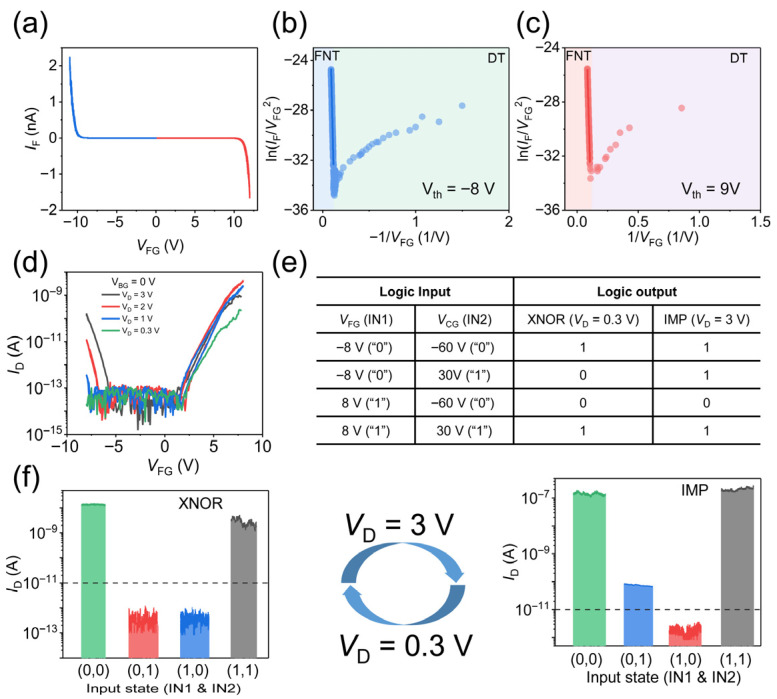
Reconfigurable logic gate operation between XNOR and IMP. (**a**) Current-voltage (I–V) tunneling characteristics for WSe_2_/h-BN/Gr heterostructure; (**b**,**c**) the ln(I_F_/V^2^_FG_) versus 1/V_FG_ curves under negative and positive floating-gate voltages; (**d**) transfer curves under the modulation of floating gate at various V_D_; (**e**) truth table for the reconfigurable logic gates XNOR and IMP; (**f**) transition between XNOR and IMP logic gates.

To gain deeper insight into the mechanism of electron tunneling, Figure 4a presents the tunneling current versus voltage characteristics for the WSe_2_/h-BN/Gr heterostructure, with the corresponding test setup shown in Figure S2. As observed, the tunneling current remains low at small floating-gate voltages. When the voltage exceeds a certain threshold, the current exhibits a sharp exponential increase. This behavior is attributed to a transition in the tunneling mechanism: direct tunneling (DT) dominates at lower voltages, while Fowler–Nordheim tunneling (FNT) becomes operative above the threshold voltage. The relationship between current density (*J*) and electric field (*E*) for direct tunneling and FN tunneling is given below:

(1) $$J_{DT}=A\exp\left[\beta E_{DT}^{\frac{1}{2}}\right]$$

(2) $$J_{FNT}=AE_{FNT}^{2}\exp\left[\frac{-B}{E_{FNT}}\right]$$

where A and B are constants [44,45]. It can be derived from the equations that for direct tunneling:$\;\ln\left(I_{DT}\right)\;\propto \;\sqrt{V_{DT}}$ and for Fowler–Nordheim tunneling: $\ln\left(\frac{I_{FNT}}{V_{FNT}^{2}}\right)\;\propto \;\frac{-1}{V_{FNT}}$. Figure 4b,c presents the plots of $\ln\left(\frac{I_{F}}{V_{FG}^{2}}\right)\;$versus $\frac{-1}{V_{FG}}$ for backward and forward sweeps of the floating-gate voltage. Figure S3 presents the plots of $\ln\left(I_{F}\right)\;$versus $\sqrt{\left|V_{FG}\right|}$ for backward and forward sweeps of the floating-gate voltage. From these curves, the threshold voltages for FNT are extracted to be approximately 9 V (forward sweep) and −8 V (reverse sweep), respectively. The experimentally observed onset voltages of −8 V and 9 V for our 11.5 nm-thick h-BN layer are in good agreement with the theoretical expectation for FN tunneling, further supporting that the high-bias transport is governed by this mechanism. The FN fit exhibits high linearity, as evidenced by the coefficient of determination (R^2^ > 0.99). From the slope, we extracted electron and hole tunneling barriers of 3.3 eV and 2.9 eV, respectively. This asymmetry stems from the difference in the energy barriers that carriers must overcome: during the forward sweep, the primary barrier is at the Gr/h-BN interface, whereas during the reverse sweep, it is at the WSe_2_/h-BN interface. The distinct heights of these two interfaces account for the observed difference in threshold voltages. Since a lower threshold voltage is required for tunneling in the negative FG direction, our work employs a drain voltage of 3 V combined with an FG voltage of −8 V to implement the IMP logic function, rather than using a drain voltage of −3 V with an FG voltage of 8 V.

Figure 4d shows the source-drain current as a function of the FG voltage under different source-drain biases, with the back gate grounded. It clearly demonstrates that a higher *V*_D_ lowers the FG voltage needed to trigger FNT. Notably, the current remains minimal before FNT occurs because the capacitive coupling effect dominates the carrier redistribution process. By exploiting the transition of the dominant operating mechanism between capacitive coupling and electron tunneling, the reconfigurable logic functions of XNOR and IMP are achieved, and the corresponding truth table is presented in Figure 4e. Here, −8 V and 8 V are used to represent the logic 0 and 1 for IN1, and −60 V and 30 V are used to represent the logic 0 and 1 for IN2. The logic output is defined by the channel current, and a logic output of “1” is defined as a current greater than 10^−11^ A, while a logic “0” corresponds to a current less than 10^−11^ A. Figure 4f displays the output currents measured under different combinations of input logic for XNOR and IMP logic. As can be seen from the figure, the device operates as an XNOR logic gate at *V*_D_ = 0.3 V and as an IMP logic gate at *V*_D_ = 3 V. Therefore, the logic function can be switched simply by adjusting the source-drain voltage.

In addition, we analyzed the intermediate voltage states to elucidate the gradual evolution of logic functionality and the underlying transport mechanisms. The transition between IMP and XNOR logic operations is primarily attributed to the change in output current under the (0, 1) input combination. Therefore, we analyze the intermediate states using the (0, 1) input as $V_{D}$ increases from 0.3 V to 3 V. At $V_{D}$ =0.3 V, FN tunneling is negligible, and the device operates in XNOR mode dominated by capacitive coupling. As $V_{D}$ increases beyond 1.1 V, FN tunneling begins to dominate, but the source-drain current remains below the 10^−11^ A threshold until $V_{D}$ exceeds 2 V. Consequently, in the intermediate range of 1.1 V to 2 V, the device still exhibits XNOR logic, but the operating mechanism transitions from capacitive coupling to FN tunneling. At $V_{D}$ = 3 V, the current is well above the threshold, enabling reliable IMP logic with improved noise margin.

## 4. Conclusions

In summary, we have demonstrated a dynamically reconfigurable logic device based on a WSe_2_/h-BN/Gr heterostructure. The carrier distribution in the bipolar WSe_2_ channel is simultaneously controlled by the floating-gate voltage, back-gate voltage, and source-drain voltage, where the floating-gate voltage regulates the channel regions on both sides of the electrode, while the back-gate voltage governs the central area of the channel. Under a small source-drain voltage, the channel carrier distribution is primarily governed by electrostatic modulation induced by the floating gate and the back gate, thereby achieving XNOR logic. As the source-drain voltage increases, Fowler–Nordheim tunneling between the floating gate and the drain electrode, coupled with significant capacitive coupling, becomes the dominant operational mechanism, enabling the device to execute IMP logic. The developed two-dimensional reconfigurable heterojunction offers a promising pathway toward compact, multi-functional logic elements with simplified operational control, providing a tangible advantage for future adaptive electronic systems. In future studies, the use of voltage pulses on the floating gate is expected to enable non-volatile and reconfigurable logic operations.

## Data Availability

The original contributions presented in this study are included in the article/Supplementary Materials. Further inquiries can be directed to the corresponding author.

## Supplementary Materials

The following supporting information can be downloaded at https://www.mdpi.com/article/10.3390/nano16050335/s1. Figure S1: Schematic illustration of the transport characteristic measurement circuit for the WSe2/h-BN/Gr floating gate device; Figure S2: Schematic illustration of the tunneling characterization circuit for the Au/WSe_2_/h-BN/Gr/Au heterostructure; Figure S3: The plots of $\ln\left(I_{F}\right)\;$versus $\sqrt{\left|V_{FG}\right|}$ for backward and forward sweeps of the floating-gate voltage; Figure S4: The bidirectional transfer characteristics and enlarged view of the tunneling current; Figure S5: The open-circuit current for over 100 s under conditions identical to those used for device characterization; Table S1: The mean current, standard deviation, and the maximum and minimum currents for the different input states of the IMP and XNOR logic gates.
