# Supplementary material for: Dynamically Reconfigurable XNOR/IMP Logic Based on Dual-Mechanism Operation in an Electrically Tunable Two-Dimensional Heterojunction
Source: Nanomaterials (Basel). 2026 Mar 9;16(5):335. doi: 10.3390/nano16050335 (PMC12986355; doi:10.3390/nano16050335)
Supplement: Supplementary file 1 [file nanomaterials-16-00335-s001.zip › nanomaterials-4126934-supplementary.pdf]

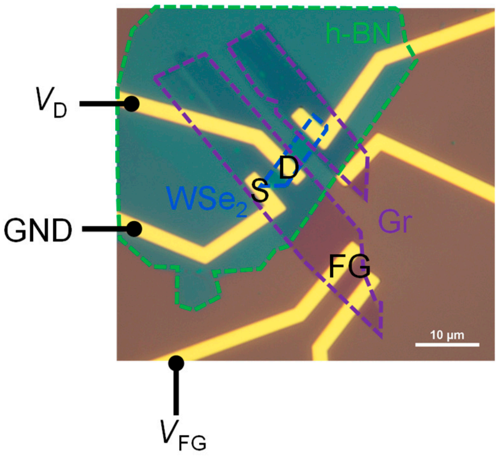

Figure S1. Schematic illustration of the transport characteristic measurement circuit for the WSe_2/h-BN/Gr floating gate device.

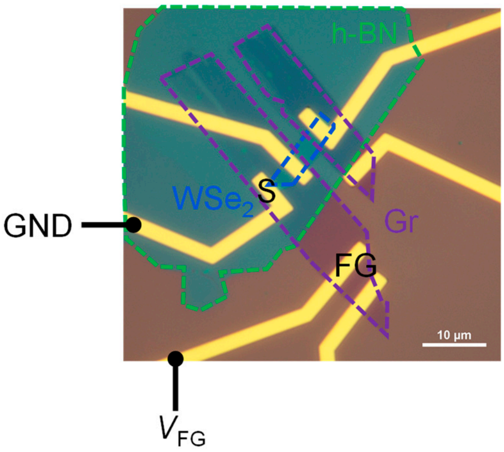

Figure S2. Schematic illustration of the tunneling characterization circuit for the Au/WSe_2/h-BN/Gr/Au heterostructure.

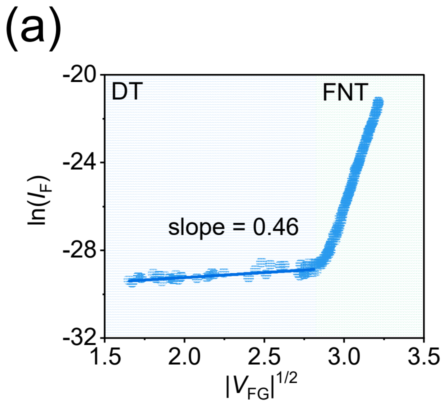

Figure S3. The plots of $\ln(I_F)$ versus $\sqrt{|V_{FG}|}$ for backward (a) and forward (b) sweeps of the floating-gate voltage.

The extracted slopes from the direct tunneling regime (0.46 and 0.07 for backward and forward sweeps) reflect the rate at which the tunneling current increases with voltage in the DT region, but we note that these values are primarily used for qualitative comparison rather than for extracting specific physical parameters.

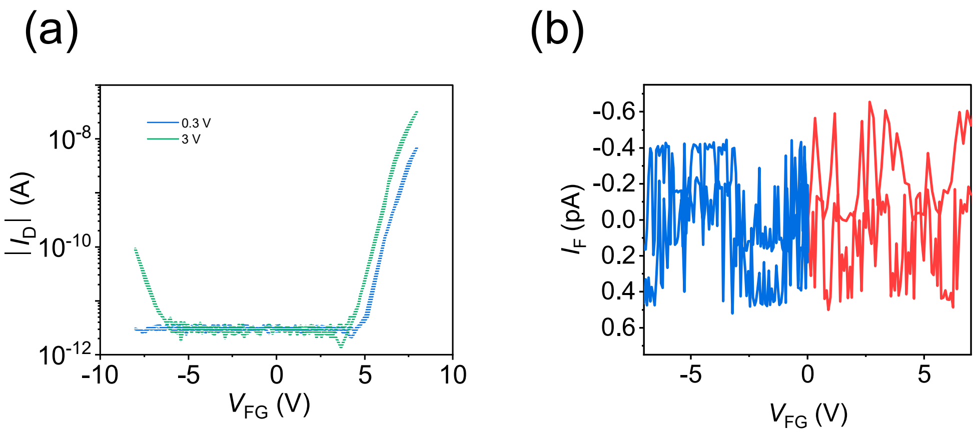

Figure S4. The bidirectional transfer characteristics and enlarged view of the tunneling current. (a) The bidirectional transfer characteristics under the modification of V_{FG} under 0.3 V and 3 V bias; (b) The enlarged view of the tunneling current from -8 V to 8 V in Figure 4a.

To characterize the defect states in our device, we have added the bidirectional transfer characteristics under the modification of V_{FG} in Figure S4.a. As shown in the figure, the transfer curves exhibit a negligible hysteresis window across the entire scanned voltage range, which indicates the high-quality of WSe_2/h-BN/Gr heterostructure and negligible charge trapping/de-trapping on the interfaces. In addition, we have included an enlarged view of the tunneling current characteristics from -8 V to 8 V in Figure S4.b. The data demonstrates that the current remains consistently at the off-state level (on the order of pA) across this voltage range, indicating a low bulk trap density in the h-BN layer. Since trap-assisted tunneling would typically yield measurable current even at moderate electric fields, this observation demonstrates that trap-assisted tunneling is not expected to play a major role.

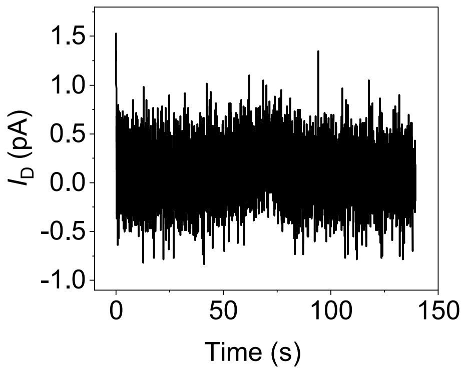

Figure S5. The open-circuit current for over 100 seconds under conditions identical to those used for device characterization.

As shown in Figure S5, the acquired data exhibit a mean value of 0.1 pA and a standard deviation of 0.28 pA, corresponding to an RMS noise floor of approximately 0.28 pA. The variance is calculated to be 7.72 × 10^{-26} A^2. Over the measurement period, the maximum and minimum current excursions are 1.53 pA and -0.84 pA, respectively, yielding a peak-to-peak noise of 2.36 pA. A signal exceeding the mean by more than three standard deviations ($\mu + 3\sigma = 0.94$ pA) can be considered genuine rather than noise.

Table S1. The mean current, standard deviation, and the maximum and minimum currents for the different input states of the IMP and XNOR logic gates.

			Mean current μ	Standard deviation σ	Max current	Min current
IMP	Logic 0	(1,0)	1.78 pA	0.53 pA	3.60 pA	-0.6 pA
	Logic 1	(0,0)	203 nA	22.3 nA	273 nA	161 nA
		(0,1)	75.1 pA	4.72 pA	86.7 pA	66.1 pA
		(1,1)	142 nA	14.6 nA	189 nA	107 nA
XNOR	Logic 0	(0,1)	0.02 pA	0.30 pA	1.04 pA	-0.79 pA
		(1,0)	-0.02 pA	0.33 pA	1.18 pA	-1.03 pA
	Logic 1	(0,0)	2.72 nA	0.85 nA	6.24 nA	1.16 nA
		(1,1)	13.5 nA	0.38 nA	14.7 nA	12.2 nA

For the IMP logic operation, the logic "0" state exhibits a mean current of 1.78 pA with a standard deviation of 0.53 pA and a maximum value of 3.6 pA. For logic "1", the input combination (0, 1) represents the worst-case scenario with the smallest output current, yet still shows a mean current of 75.1 pA with a standard deviation of 4.72 pA and a minimum value of 66.1 pA. The selected threshold of 10^{-11} A lies well outside both the $\mu + 3\sigma$ range of logic "0" (1.78 + 3 × 0.53 = 3.37 pA) and the $\mu - 3\sigma$ range of logic "1" (75.1 - 3 × 4.72 = 60.94 pA), ensuring reliable discrimination between the two logic states. The minimum signal-to-noise ratio (SNR) between logic "0" and logic "1" is calculated to be 261, based on the formula $SNR = \frac{\mu_1 - \mu_0}{\sigma_{noise}}$. μ_1 and μ_0 are the mean currents of logic "1" and logic "0", respectively, and σ_{noise} is the standard deviation of the noise floor. This high SNR value further confirms that the two logic states are well separated and can be distinguished with high reliability. For the XNOR logic operation, the logic "0" state exhibits a mean current on the order of 10^{-14} A, while the logic "1" state corresponds to currents above 1 nA. The logic "0" state exhibits an extremely low mean current because the current itself is extremely low, approaching the measurement limit of the system. As a result, the measured values fluctuate between positive and negative, leading to a small average. Nevertheless, this does not compromise reliable logic state discrimination. As a result, a threshold of 10^{-11} A ensures reliable differentiation between the two logic states, and the minimum SNR is 45428. The above analysis for two representative logic gates confirms robust separation using the universal drain-current threshold of 10^{-11} A.
